# Assessing Website Pharmacy Drug Quality: Safer Than You Think?

**DOI:** 10.1371/journal.pone.0012199

**Published:** 2010-08-13

**Authors:** Roger Bate, Kimberly Hess

**Affiliations:** 1 American Enterprise Institute, Washington, District of Columbia, United States of America; 2 Africa Fighting Malaria, Washington, District of Columbia, United States of America; University of California San Francisco, United States of America

## Abstract

**Background:**

Internet-sourced drugs are often considered suspect. The World Health Organization reports that drugs from websites that conceal their physical address are counterfeit in over 50 percent of cases; the U.S. Food and Drug Administration (FDA) works with the National Association of Boards of Pharmacy (NABP) to regularly update a list of websites likely to sell drugs that are illegal or of questionable quality.

**Methods and Findings:**

This study examines drug purchasing over the Internet, by comparing the sales of five popular drugs from a selection of websites stratified by NABP or other ratings. The drugs were assessed for price, conditions of purchase, and basic quality. Prices and conditions of purchase varied widely. Some websites advertised single pills while others only permitted the purchase of large quantities. Not all websites delivered the exact drugs ordered, some delivered no drugs at all; many websites shipped from multiple international locations, and from locations that were different from those advertised on the websites. All drug samples were tested against approved U.S. brand formulations using Raman spectrometry. Many (17) websites substituted drugs, often in different formulations from the brands requested. These drugs, some of which were probably generics or perhaps non-bioequivalent copy versions, could not be assessed accurately. Of those drugs that could be assessed, none failed from “approved”, “legally compliant” or “not recommended” websites (0 out of 86), whereas 8.6% (3 out of 35) failed from “highly not recommended” and unidentifiable websites.

**Conclusions:**

Of those drugs that could be assessed, all except Viagra® passed spectrometry testing. Of those that failed, few could be identified either by a country of manufacture listed on the packaging, or by the physical location of the website pharmacy. If confirmed by future studies on other drug samples, then U.S. consumers should be able to reduce their risk by relying on credentialing agencies recommended lists and by using common sense when examining packaging and pills.

## Introduction

Spending for prescription drugs in the United States hit $216.7 billion in 2006, more than triple the $40.3 billion spent in 1990 [1]. While the lion's share is still spent in large chain stores like CVS Caremark or RiteAid, consumers, some probably uninsured, on a quest for cheaper drugs or drugs off-prescription are embracing alternative distribution channels–and are increasingly buying over the Internet [2], [3].

In 2009, 30 percent of prescription drug users reported buying drugs online or through the mail in the previous 12 months, a 9 percent increase over the number who said so in 2008, according to the Deloitte Center for Health Solutions [4]. Reluctant to acknowledge purchasing so-called “lifestyle” drugs like painkillers or those used to treat erectile dysfunction or depression—among the most popular drugs sold over the Internet—consumers may underreport their online purchasing behavior. U.S. fraud prevention and brand protection firm Mark Monitor estimates the total size of the online market at $12 billion [5].

Website pharmacies are diverse. They include licensed U.S. pharmacies accredited by the National Association of Boards of Pharmacy (NABP), and licensed foreign pharmacies approved by the private credentialing group PharmacyChecker.com, as well as outfits based in undisclosed international locations, willing to illegally divert and distribute drugs without a prescription. Non-credentialed website pharmacies often sell drugs after consumers complete a brief medical questionnaire, allegedly reviewed by a physician at the website pharmacy without requiring a prescription, whereas licensed pharmacies always require a prescription from a physician.

Until recently, much of the academic literature focused on understanding which drugs—brand and generic—Americans could buy over the Internet, under what conditions—on or off-prescription—and at what prices. The quality of drugs available on the Internet was a secondary consideration, if it was considered at all [6]. With incidents of poor drug quality widely reported in the media, and with policymakers considering legislation that would allow Americans to buy drugs directly from Canada, researchers have begun to shift their focus.

Purchasing drugs over the Internet can offer significant benefits, including, as the FDA acknowledges, a “convenient, private, way to obtain needed medications, sometimes at more affordable prices.” The elderly, infirm, or geographically isolated may be able to obtain prescriptions more quickly and easily. But drugs purchased from unverified website pharmacies without a valid prescription can be dangerous.

According to a 2004 Wall Street Journal/Harris Interactive poll, most Americans (61 percent) think online drug purchasing can be dangerous, but nearly one-in-four (23 percent) say they “aren't sure” whether drugs purchased online are more or less dangerous than drugs purchased through traditional pharmacies [7]. Even though federal law technically prohibits the importation of drugs from overseas except under special circumstances, 4% of prescription drug users in the United States said in 2009 that they bought from a foreign source, and 20% of all consumers said they would likely buy from a source outside the United States if they could save 50% or more in price [4].

The authors aimed to assess the quality of prescription drugs that could be purchased over the Internet, and under what conditions. In order to compare the quality of drugs procured from websites subject to different levels of regulatory supervision, the authors intended to stratify the sample into four groups: “approved,” “legally compliant,” “not recommended,” and “highly not recommended” (See Appendix S1).

## Materials and Methods

The authors identified drugs most likely to be purchased by American consumers in several drug classes by cross-tabulating consumer self-reports [7] with industry data, including lists of the most-popular online drug searches from licit website pharmacies and IMS's list of the top 10 products “most often prescribed” in the United States in 2007. The five drugs selected for purchase were (in order of selection priority):

Lipitor® 10mg (atorvastatin calcium) a synthetic lipid-lowering agent to reduce cholesterol, manufactured by Pfizer Inc.
Viagra® 100mg (sildenafil citrate) an oral therapy for erectile dysfunction, manufactured by Pfizer Inc.
Celebrex® 200mg (celecoxib) a nonsteroidal anti-inflammatory drug for treatment of arthritis, manufactured by Pfizer Inc.
Nexium® 40mg (esomeprazole magnesium) a proton pump inhibitor for treatment of Gastroesophageal Reflux Disease, manufactured by AstraZeneca Pharmaceuticals LP
Zoloft® 100mg (sertraline HCl) a selective serotonin reuptake inhibitor for treatment of depression, manufactured by Pfizer Inc.

The dosages chosen were the most popular among identified websites and after consultation with Joseph Moody, MD, the physician advising this study. With the approval of his state health board, Dr. Moody provided prescriptions for the drugs. The prescriptions were for the established dosages but in varying quantities. A uniform homogeneous database of samples from website pharmacies was not possible since many websites only sell in prohibitively large (and expensive) quantities, do not sell all five brand-name versions of the drugs, or do not sell the drugs in the required dosages. While only Zoloft® is no longer under patent in the United States, websites sourcing drugs from overseas may try to supply generic or copy versions of the other drugs, which may be legally produced for domestic consumption in other parts of the world (although illegal if sold in the United States). During the procurement process, the authors always instructed website pharmacies to provide brand-name drugs, and did not procure from websites where only generic versions were available.

Once the most popular dosages were identified, reference standards were established for the chosen handheld Raman spectrometer. The spectrometer created a detailed spectral “fingerprint” for each reference standard, which was then compared against spectral readings from drugs procured over the Internet. To create the reference standards, genuine samples provided via prescription by a national pharmacy chain (West Lafayette, IN, USA) were analyzed using the Raman spectrometer and cross-checked against a second lot from a separate pharmacy to verify consistency and determine method robustness. In cases where it appeared slight lot-to-lot variation was present (as in the case for Lipitor® coating thickness), a reference spectrum from both lots was included in the Raman spectroscopic method. In all cases, the two lots of drugs matched well and it was deemed that they were representative samples of authentic products.

Drugs were ordered in January, February, and then again in September 2009 from website pharmacies identified using Google and Yahoo! search criteria and the NABP list of approved and not recommended websites (See Appendix S1), as well as examination of spam emails sent to the authors and those caught in the spam filters of their organizations. Attempts to purchase from some websites were unsuccessful. Two website pharmacies from the NABP-approved list returned prescriptions; three websites from the not recommended list would not accept payment; and in two cases, ordered drugs from highly not recommended websites were never received. While every reasonable effort was made to procure drugs from each website, this was not always possible. The lead author attempted to procure drugs from websites experiencing problems three times before moving on to the next website.

Fourteen of the drug packages received could not be linked directly to a website because multiple purchases were ‘active’ at the same time, and the packaging was not identifiable. The purchases were not made in a linear fashion – ordering from one website and not ordering from the next until the first was received.

The authors assessed drug quality using Raman spectrometry. Numerous studies have demonstrated that Raman spectrometry is a quick, reliable and cost-effective way for non-specialists to differentiate between genuine and counterfeit drugs [8]–[11]. To ascertain the nature, and not just the spectra, of all compounds in a given drug, including impurities and degradation products as well as active ingredients, high-performance liquid chromatography (HPLC), considered the current gold standard analytical method in drug analysis, would be required. HPLC requires sophisticated sample preparation that is expensive and time consuming and requires trained chemists for analysis and interpretation of results. Given that the aim of this study was to authenticate a finished product (rather than its individual components), comparison with a known HPLC standard was unnecessary. The authors used a handheld Raman spectrometer, the TruScan by Ahura Scientific (Wilmington, MA), on loan for the duration of the study. One necessity, and potential limitation, of spectrometers is that they require exact reference standards, obtained by scanning each separate brand with the same formulation for calibration. This means that a drug substituted for the branded version would record likely as a failure (since the excipients could be different, yielding different spectra, between two equally effective drugs). For this reason, generic substitutes were not sought from websites for this study.

While a pass identifies a good quality drug, a “failure,” as assessed by the authors, does not mean that a given drug is necessarily of low quality. The spectrometer recorded a “failure” if a sampled drug was spectroscopically inconsistent with the reference standard; under this metric, both copy versions and FDA-approved bioequivalent generics of the chosen drugs may fail, because while they must contain the same quantities of active ingredient, they often contain different binding agents (excipients) in different concentrations. The spectrum created by the spectrometer is for the total sample formulation, not only the active ingredient.

Four samples from each drug from each website pharmacy were tested; additional samples were tested when there were failures, with the more positive results being recorded. Five websites only sent three tablets of Viagra® each, all of which were tested.

In order to compare prices of drugs with the same formulations, purchased in the same quantities, the authors identified prices posted on the website or quoted by a pharmacy representative over the phone in early May 2009. If the formulation was not available in the same quantity, the authors selected the closest quantity available. Prices were calculated as “stand-alone” orders; that is, shipping expenses were not amortized across the entire five-drug order. The authors also identified prices of the five drugs sold at physical-location pharmacies in the Washington, D.C. area. Six pharmacies were selected as the closest within a 0.5 mile radius of the lead author's home address using Google Maps. No more than one of each chain pharmacy was selected.

## Results

**Figure 1:**
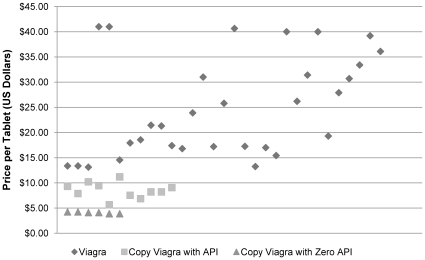
Viagra® and its copies by price in US$ (including 14 samples of suspected Viagra® whose identity could not be confirmed). “Copy Viagra with API” refers to samples which are copies of Viagra® (it was not established whether they were bioequivalent copies - generics) and, from spectrographic analysis, which contain the correct active pharmaceutical ingredient (API) – sildenafil citrate. “Copy Viagra with Zero API” refers to samples which are copies of Viagra® that do not contain any sildenafil citrate.

The authors received drugs from 55 website pharmacies, among them six “approved,” ten “legally compliant,” ten “not recommended,” fifteen “highly not recommended,” and fourteen that were “not recommended” or “highly not recommended” but could not be identified by name because the packaging provided could not be perfectly matched with the pre-orders. A total of 152 drug orders were received, including 25 Celebrex®, 25 Lipitor®, 22 Nexium®, 50 Viagra®, 28 Zoloft® and two unknowns from two unidentifiable websites. Of these, samples from 121 drug orders were tested using Raman spectrometry. Nexium® tablets from eleven websites and Zoloft® capsules from five websites could not be assessed because the authors' spectrometry protocol was established with reference standards for Nexium® capsules and Zoloft® tablets (the typical drug formulations sold in the United States). Additionally, one order of “sertraline HCl” tablets and three orders of “Daxid®” tablets were received in place of Zoloft® and could not be assessed since reference standards were not available. Lastly, blue tablets shaped like Viagra®, which were not labeled “Viagra®” and not labeled as being manufactured by Pfizer, were received from three “highly not recommended” websites and six unidentifiable websites; they were not assessed in the main analysis since their identity could not be confirmed (these samples are included in the 50 Viagra® drug orders received above, and are included in Figure 1 for price and quality of Viagra® and its copies). Two unknown drugs accompanied two of the suspected Viagra® orders (it was presumed that these unknowns were copies of the erectile dysfunction drug Cialis®, given their shape, size and coloring) and were not assessed since their identity could not be confirmed.

Seventy samples were tested from January to April 2009, and 51 samples were tested in April 2010. 2.5% (3/121) of tested samples failed Raman spectrometry: 0% of Celebrex® (0/25), 0% of Lipitor® (0/25), 0% of Nexium® (0/11), 7.3% of Viagra® (3/41), and 0% of Zoloft® (0/19) (See Table 1).

**Table 1:** Spectrometry testing results by drug type and website pharmacy classification.

	Website classification	Number received	Number in testable form	Percent that failed testing
Lipitor®	NABP approved^i^	6	6	0%
	Legally compliant^ii^	10	10	0%
	Not recommendediii	5	5	0%
	Highly not recommended^iv^	4	4	0%
	Combined Total	25	25	0%
Viagra®	NABP approved	6	6	0%
	Legally compliant	10	10	0%
	Not recommended	5	5	0%
	Highly not recommended	15	12	8.3% (1/12)
	Unidentifiable	14	8	25% (2/8)
	Combined Total	50	41	7.3% (3/41)
Celebrex®	NABP approved	6	6	0%
	Legally compliant	10	10	0%
	Not recommended	5	5	0%
	Highly not recommended	4	4	0%
	Combined Total	25	25	0%
Nexium®	NABP approved	6	6	0%
	Legally compliant	10	3	0%
	Not recommended	2	0	
	Highly not recommended	4	2	0%
	Combined Total	22	11	0%
Zoloft®	NABP approved	6	5	0%
	Legally compliant	9	6	0%
	Not recommended	6	3	0%
	Highly not recommended	7	5	0%
	Combined Total	28	19	0%
Unknown	Unidentifiable	2	0	
TOTAL	NABP approved	30	29	0%
	Legally compliant	49	39	0%
	Not recommended	23	18	0%
	Highly not recommended	34	27	3.7% (1/27)
	Unidentifiable	16	8	25% (2/8)
	Combined Total	152	121	2.5% (3/121)

i “Approved”: have been accredited by the U.S. NABP.

ii “Legally compliant”: have not been accredited by NABP, but nor are they listed on NABP's “not recommended” list. PharmacyChecker, an independent group not affiliated with any given pharmacy, indicates that they are in compliance with the laws in the country in which they are registered.

iii “Not recommended”: approved by PharmacyChecker, but “not recommended” by NABP.

iv “Highly not recommended”: “not recommended” by NABP and unlisted or “not approved” by PharmacyChecker.

Only brand-name drugs were ordered in this study; however, 15 website pharmacies failed to comply with instructions and sent copy versions in place of the brand-name drug. The authors did not attempt to verify the authenticity or quality of these substitutes since reference standards were not available. Previous attempts to obtain samples from various companies were only partly successful; the authors were concerned that reference samples would not be received from all companies in a timely manner, potentially biasing results. It is possible that drug substitution may have occurred unbeknownst to the authors, i.e. the drugs were not labeled as such, and as a result they may have failed testing due to different excipients as explained above.

Of the website pharmacies accredited by NABP, only one provided a drug which was not in testable form; in this case, the bottle was labeled “Sertraline HCl 100mg.”

Eight “legally compliant,” “not recommended,” or “highly not recommended” website pharmacies sent Nexium® tablets instead of capsules, allegedly produced for AstraZeneca in Turkey (5 orders), Canada (2 orders), and Sweden (1 order). By comparing the Raman spectra of the Nexium® tablets against the spectra of the Nexium® capsules, the authors determined that all of the Nexium® tablets appeared to contain active ingredient. Additional follow-up of samples from two of the Nexium® tablet orders with AstraZeneca's Global Quality Operations identified the tablets and their batch numbers as consistent with genuine product packaged for AstraZeneca in Turkey.

Three orders of tablets labeled “Neksium®” instead of Nexium® were received from “legally compliant” or “not recommended” website pharmacies. They were allegedly manufactured by a company for AstraZeneca India in Bangalore. The tested samples had spectra unlike the Nexium® capsules or Nexium® tablets, and did not appear to contain the main Nexium® active ingredient, esomeprazole. A high level of fluorescence was associated with the individual spectra of these tablets which precluded the automated analysis. The packaging indicated that Neksium® is “For Sale in India and Nepal Only.” One website pharmacy attempted to cover up this text by placing a sticker “To be dispensed against doctor's prescription only” on top of it. The authors informed AstraZeneca about this potentially counterfeited drug and, as a result, AstraZeneca launched an internal investigation. After careful consideration of samples from two of the Neksium® orders, a report was returned to the authors indicating the tablets and their batch numbers were consistent with product manufactured and packaged for AstraZeneca India by a third party contractor.

Three “legally compliant” or “not recommended” website pharmacies sent “Daxid®” tablets in place of Zoloft® tablets, which were not in testable form but passed spectrometry testing when tested against the Zoloft® reference standard. The packaging indicated that Daxid® is “For Sale in India Only” and is manufactured by Pfizer Limited in India. All website pharmacies attempted to cover up this text by placing either a blank sticker or one that read “To be dispensed against doctor's prescription only” on top of it.

Of the 55 website pharmacies sampled, and of those drugs which could be tested against established reference standards, 0% (0/29) of drugs from “approved” websites failed, 0% (0/39) of drugs from “legally compliant” websites failed, 0% (0/18) of drugs from “not recommended” websites failed, 3.7% (1/27) of drugs from “highly not recommended” websites failed, and 25% (2/8) of drugs from websites that were either “not recommended” or “highly not recommended” but could not be identified by name failed (See Table 1). All drugs purchased from the “approved” and “legally compliant” website pharmacies were accounted for, whereas the latter classifications had websites which may not have delivered drugs, or had delivered drugs in unidentifiable packaging and were not identifiable in other ways.

“Highly not recommended” website pharmacies tended to offer fewer types of drugs than the other website classifications. Of these websites, only four delivered any of the five drugs other than Viagra®. In two cases, Viagra® and Nexium® were initially ordered, but only Viagra® was delivered. In the first instance, Nexium® was crossed out on the package receipt but the author's account was charged for the missing drug, in the second, the author's account was charged for the Viagra® but not the Nexium®.

The authors assessed the country of manufacture, where possible. Many websites, particularly NABP-approved websites, like most U.S. retail pharmacies, sent drugs packaged in orange, cylindrical “pill pots,” which often did not list a country of manufacture. Of the 152 drug orders received 43% (65) came from outside of North America and 16% (24) from within North America (Canada 8, United States 16). The remaining 41% (63) were of unknown origin or could not be assessed.

Drugs were manufactured in Italy (1.5 samples), Germany (0.5 sample), Australia (6 sample), Australia/New Zealand (21 samples; both countries were listed on the same package; a phone call to the pharmacy could not clarify where these drugs were manufactured), India (12 samples), Sweden (1 sample), Turkey (23 samples), Canada (8 samples) and the United States (16 samples). For one of the drug orders, half of the packages received listed Italy as the country of manufacture and the other half listed Germany. Of the remaining 63 drug orders, it is likely that many were manufactured in the U.S., given their source (NABP-approved websites), appearance, and formulations. Some of the drugs with an unknown country of manufacture, however, likely came from elsewhere: drugs that were probably copy versions of Viagra® arrived with postmarks from India, for example.

Half of the samples with packaging that said they were manufactured in Canada (4 of 8), all of the samples from India (12 of 12), the single sample from Sweden (1 sample), 13 of the 23 samples from Turkey, and 1 of 16 samples from the United States were not in testable form. Of the drugs that were in testable form, only one that listed a country of manufacture (in this case, Viagra® tablets that were allegedly manufactured in the United States) failed spectrometry testing.

### Problems of Provenance & Packaging

Many drugs, including some that did not fail spectrometry testing, had worrying irregularities in provenance or packaging. One website pharmacy claimed to be based in Ontario, Canada, but the receipts included in the packages stated that the drugs had been shipped from Australia and India, as well as the United States. The return addresses on the packages indicated that they had passed through Vanuatu, Germany, or Santa Ana, California. Four of the five drugs (with the exception of Neksium®, which was not in testable form) purchased from this website pharmacy passed spectrometry testing.

In 12 cases, drugs were shipped from a different location than was indicated on the website. Three claimed to be U.S. or Canada-based and advertised prices in dollars on their website, yet charged in Chinese or Indian currency. One website pharmacy described itself as an “off-shore company based in Cyprus,” but listed a contact address in British Columbia. The authors were not able to determine where exactly the funds were deducted from for the transaction. In reviewing statements from the bank account used in this study, the initial transfer was sent to Panama, not a known location for the company. Viagra® ordered from this website arrived wrapped in aluminum foil, with a postmark from Shanghai. Labeled as “Pfizer Inc. USA” Viagra®, all four tablets tested failed Raman spectrometry. The shipping envelope and drug packaging was similar to a reported port security seizure one of the authors had seen at a public presentation by a Pfizer security expert.

Eleven website pharmacies shipped international versions of brand-name drugs. One website pharmacy provided Zoloft® capsules that were bright orange in a bottle labeled as “Pfizer Canada.” Another website, based in Vanuatu, shipped Celebrex®, Lipitor®, and Zoloft® marked “Pfizer Australia” – all of which passed Raman spectrometry testing. Another website pharmacy claimed to be based in British Columbia, Canada, but the drug orders arrived with postmarks from the Deutsche Post, with a Swiss Post Declaration and Customs Authorities Form, and a Zurich Airport Label. The drugs received were allegedly manufactured by three separate drug companies. Another website pharmacy sent an order of loose tablets in a plastic bag with packaging and information inserts that could not be connected with any of the initial purchase websites. In this case, the drug order arrived with a return address for Vienna, Austria. The tablets were blue in color, shaped like Viagra® and stamped with “Pfizer” - all tested samples failed Raman spectrometry.

### The Purchasing Experience

Four website pharmacies could not supply all the drugs they claimed to offer. One website claimed to have “sold out” of Lipitor® when the authors attempted to procure it. Six websites were portals to many other websites, many of which crashed after the lead author submitted the medical questionnaire that accompanies many websites. When this happened, drugs could not be procured.

Thirty-seven website pharmacies offered prescription drugs without requiring an original prescription. All of the NABP-approved and “legally compliant” websites, except one, either demanded original prescriptions or (for two websites) accepted faxed prescriptions but followed-up with the prescribing physician to establish provenance. These website pharmacies appeared to focus on building a long-term relationship with the consumer. Indeed, seven of the 18 websites described by NABP as “recommended” would not even sell prescription drugs to an individual unless he or she was connected with a medical insurer.

All of the “not recommended” and “highly not recommended” website pharmacies claiming to require prescriptions accepted faxed or emailed copies without contacting the prescribing physician to confirm. One website openly advertised the provision of drugs off prescription. Other website pharmacies offered to supply drugs after the consumer filled out an online evaluative questionnaire, which varied in length and complexity. Most of the “not recommended” and “highly not recommended” website questionnaires were very basic, although five took at least 10 minutes to complete and asked questions relevant to the conditions and contraindications associated with the drugs.

### “Cheap” Drug Websites: Buyer Beware?

There was wide price variation among the procured drugs. Among purchased drugs, which had some variation in formulation and quantity, Viagra® was, on average and within each website classification, the most expensive drug, and also the drug with the greatest range in price. Prices per tablet for Viagra® ranged from $13.12 to $41.00 per tablet, including shipping expenses and other expenses (one website charged a processing fee and another website charged for a pill splitter) amortized across the entire order. This compared to ranges between $1.14 and $19.40 per tablet/capsule for Nexium®, $0.91 to $7.07 for Lipitor®, $1 to $4.63 for Zoloft®, and $1.24 to $4.27 for Celebrex®.

In general, larger orders tended to have lower per-tablet/per-capsule prices, and were more prevalent among website pharmacies that had not been approved by NABP. In order to determine whether the difference in price observed was due to order size, the authors assessed the prices offered for all of the drugs in consistent formulations (insofar as was possible; not all websites offered drugs in the same, consistent quantities) (See Table 2). In this case, orders were considered “stand-alone” and shipping prices were not amortized across the entire order. Average prices for Nexium®, Lipitor® and Zoloft® at NABP-approved websites were slightly more expensive than the other three classifications. The average price of Celebrex® from NABP-approved websites was more than twice that of the other website classifications. Viagra® offered from non-credentialed websites, was on average far more expensive than Viagra® from credentialed websites. For all of the drugs except Viagra®, prices were most expensive at physical-location pharmacies (See Table 2).

**Table 2:** Average price per tablet/capsule (minimum-maximum) of drugs with as consistent formulations and quantities as possible (not purchased).

Website Classification (number of websites*)	Celebrex® 200mg×90	Lipitor® 10mg×90	Nexium® 40mg×28	Viagra® 100mg×4**	Zoloft® 100mg×100
NABP approved (6)	$3.89 (3.54–4.06)	$2.98 (2.71–2.98)	$5.74 (5.07–6.20)	$17.21 (13.94–20.55)	$3.68 (3.12–4.32)
Legally compliant (10)	$1.91 (1.33–3.84)	$1.72 (1.00–2.89)	$3.11 (1.43–5.62)	$14.94 (12.38–17.49)	$2.11 (1.05–3.53)
Not recommended (10)	$1.76 (1.72–1.84)	$1.58 (1.09–1.88)	$3.08 (2.50–3.75)	$20.5 (15.00–36.00)	$1.56 (1.08–2.21)
Highly not recommended (15)	$1.63 (1.58–1.69)	$1.69 (1.61–1.76)	$5.56 (2.71–7.03)	$23.95 (17.25–33.65)	$2.70 (1.60–3.79)
	Celebrex® 200mg×30	Lipitor® 10mg×30	Nexium® 40mg×30	Viagra® 100mg×4	Zoloft® 100mg×30
Physical-location pharmacy*** (5)	$4.50 (4.00–5.07)	$3.44 (3.00–3.87)	$6.26 (5.40–6.83)	$17.75 (15.58–20.35)	$4.36 (4.03–5.03)

*Not all websites offered drugs in the same, consistent quantities.

**There was greater variation in the quantities for Viagra® than for the other drugs. Quantities ranged from 3 tablets to 10 tablets.

***“Physical-location pharmacy”: Pharmacies were selected as the closest within a 0.5 mile radius of the lead author's home address (using Google Maps). No more than one of each chain pharmacy was selected. In addition, the authors included a nearby Wal-Mart store. One pharmacist refused to provide price information.

If sellers of diverted drugs have relatively low transit and transaction costs, they can profit handsomely. Neksium® procured from one website for $83 ($2.96/tablet) included a label on the box that indicated it could be sold for no more than INR 6.73/tablet in India (approximately $0.14/tablet depending on the exchange rate), a mark-up of more than 2000%. For an additional twenty drugs whose packages included list prices from countries other than the United States, mark-ups ranged from −36% to more than 1400% (See Table 3).

**Table 3:** Diverted drug mark-ups: Price per tablet/capsule.

Drug Received	Price US$	Other Price	% Mark-Up (difference price/other)
Celebrex® 90×200mg	$1.22	EU 1.43 ( = $1.91)	−36%
Lipitor® 90×10mg	$0.89	TRY 1,07 ( = $0.67)	33%
Lipitor® 30×10mg	$1.67	TRY 1,07 ( = $0.67)	149%
Lipitor® 30×10mg	$1.83	TRY 1,08 ( = $0.67)	173%
Lipitor® 90×10mg	$1.22	TRY 1,08 ( = $0.67)	82%
Lipitor® 90×10mg	$1.37	TRY 1,08 ( = $0.67)	104%
Neksium® 28×40mg	$1.07	INR 6.81 ( = $0.14)	664%
Nexium® 28×40mg	$2.68	TRY 2,27 ( = $1.45)	85%
Nexium® 28×40mg	$3.39	TRY 2,27 ( = $1.45)	134%
Neksium® 28×40mg	$2.96	INR 6.73 ( = $0.14)	2014%
Neksium® 28×40mg	$2.14	INR 6.81 ( = $0.14)	1429%
Nexium® 28×40mg	$2.37	TRY 2,26 ( = $1.45)	63%
Nexium® 28×40mg	$2.47	TRY 2,26 ( = $1.45)	70%
Viagra® 4×100mg	$15.25	TRY 17,73 ( = $11.10)	37%
Viagra® 4×100mg	$12.50	TRY 18,6 ( = $11.89)	5%
Viagra® 8×100mg	$14.50	TRY 17,73 ( = $11.34)	28%
Zoloft® 112×100mg	$1.92	EU 1.61 ( = $2.16)	−11%
Zoloft® 100×100mg	$1.45	INR 9.08 ( = $0.18)	706%
Zoloft® 100×100mg	$0.98	INR 8.67 ( = $0.18)	444%

Since the only drug samples (in testable form) to fail testing were fake copies of Viagra® containing zero active pharmaceutical ingredient (API), the prices of the drugs that failed and those that passed were analyzed, as well as the prices of those that were copies of Viagra®, which had active ingredient but would “fail” spectrometry testing.

The results were stark; the fake Viagra® with zero API were the cheapest, followed by the Viagra® copies containing API, followed by actual Viagra®, and the differences were all statistically significant (See Figure 1).

## Discussion

Few drugs that could be tested against reference standards, failed; there was, however, some drug substitution, which limited the number of samples that could be assessed. In addition, Nexium® tablets are not approved for sale in the United States, but are common in Europe and much of the rest of the world. Since tablets have different excipients and different coatings than capsules, their spectra are slightly different. As a result, samples from each of the Nexium® tablet orders failed when tested against the Nexium® capsule reference standard. Even assuming all substitutes were bioequivalent copies (i.e. generics), risks remain for unwary purchasers of drugs over the Internet. Substandard Viagra®, almost certainly counterfeit, was procured with packaging that indicated the samples originated in China. There have been reports that many fake pharmaceuticals originate in China and India [12].

The study findings underscore the difficulty for monitoring organizations like the NABP and PharmacyChecker.com to keep their “recommended” and “not recommended” website lists up-to-date. Internet search engines *require* all advertisers and their affiliates who sell prescription drugs to be approved by PharmacyChecker.com. One website pharmacy appeared to have changed classification during the course of the study: when drugs were purchased from the website, it was within the “legally compliant” classification, listed as approved by PharmacyChecker.com, and was not listed on NABP's “not recommended” list. It later appeared on NABP's “not recommended” list. (In this study, the website was listed as “legally compliant” to reflect classification at the time of selection.) All of the drugs from this website passed spectrometry testing or were not in testable form (Neksium®).

Website pharmacies can, in principle, shut down and simply re-open under a new name. For website pharmacies based in foreign countries, the FDA and Drug Enforcement Agency have little recourse for action except to appeal to national authorities in these countries. Encouragingly, evidence suggests that some foreign governments are taking action, as the Chinese government did when it publicly blacklisted 25 websites for selling and/or advertising counterfeit and substandard products in March 2009 [13].

The variety of price mark-ups—and some mark-downs—illustrate a tiny part of the inner-workings of an illicit, global “parallel” trade in pharmaceuticals (although wholesalers are permitted to sell pharmaceuticals across borders in the European Union, such practice is illegal in the United States). The presence of a mark-down at first appears puzzling: why would a seller offer a pharmaceutical for $1.22 over the Internet when he or she theoretically could sell it for $1.91 in the European Union? It is possible that the market in the European Union may have been “flooded” with the product, thereby depressing prices. The product may have been stolen; by peddling it overseas, the seller faced less chance of detection; in a market characterized by government-imposed profit caps, heavy taxes, and other disincentives to sell the product to the highest bidder, wholesalers and/or pharmacies might well be eager to sell the product overseas.

Of course, legitimate website pharmacists may well have received drug supplies in large enough amounts to be able to offer discounted prices, but given small markups and the relatively competitive market of Europe this is unlikely to be more than a few percentage points.

While a larger sample size is needed to make broad conclusions, findings suggest that drugs procured from so-called “cheap” website pharmacies may not *always* be less expensive than more reputable websites, or at least in the case of Viagra®, even less expensive than physical-location pharmacies. Several of the “not recommended” and “highly not recommended” website pharmacies required that consumers purchase drugs in large quantities, or only permitted consumers to purchase one kind of drug at a time (each time adding processing and shipping fees), which increased the overall price even more.

The ease with which the lead author was able to procure drugs from non-credentialed websites without a prescription or by using photocopies or faxed versions of prescriptions suggests that there is ample opportunity for prescription drug abuse. The lead author was able to procure several drugs from 55 website pharmacies under the same consumer name over several months. For the websites that required prescriptions, the lead author was able to use the same prescriptions more than five times because of the lack of insistence on original versions (many website pharmacies allow consumers to fax prescriptions without contacting the prescribing physician). Not one website pharmacy checked why the lead author, a resident of Washington D.C., was using a physician in Indiana for his prescriptions (he has no obvious ties to Indiana and has never visited the state).

Regulatory agencies or associations such as the NABP or PharmacyChecker.com might consider establishing an information-sharing system among registered pharmacies that would allow pharmacists to quickly identify whether a prescription for a given drug has already been dispensed to a given individual. Such information systems already exist for physicians to track prescriptions of controlled substances in some states, but are not widely used [14].

Of course, one of the reasons consumers buy over the Internet is convenience, and often they know exactly what drugs they require. There may be frustration in dealing with increased drug delivery bureaucracy. The authors suggest that the situation established by the better known websites may be the way forward. A consumer establishes a profile with a website pharmacy, sends in an original prescription for each drug, and from then on everything can be done by email and phone, with the pharmacist dealing directly with the prescribing physician for drug refills. This also allows the patient to establish a relationship with the pharmacist to receive drug counseling and adverse effects monitoring. This provides the pharmacist and physician assurance that the patient is not abusing the drugs and is taking them safely, and it is also convenient for the patient.

Not all consumers—particularly those wishing to bypass the requirement for a doctor's prescription, however—will favor such an option. And no doubt they will shop around for the cheapest option; for their sake, one hopes they find safe products as well. Furthermore, many websites seem to price products in ways that make it more expensive than otherwise would be the case, making each drug type a separate order with delivery and other charges built in. Shopping around to avoid this should save money and over time will probably drive the more expensive sellers from the market.

### Political Discussion and Conclusions

This study demonstrates that there are many website pharmacies, including those from overseas, from which it is almost certainly safe to procure medicines; indeed, all of the pharmacies approved by both NABP and PharmacyChecker.com passed the authentication spectrometry tests undertaken in this study.

With changes to U.S. healthcare legislation and the increased push for drug importation, the debate about drug safety over the Internet is likely to intensify. The economic and ethical arguments for market segmentation, preventing wholesale importation, are strong. But few Americans care about this when they pay the highest prices globally for drugs. Some interests opposed to importation therefore claim drug buying over the Internet is dangerous, obfuscating unlicensed website pharmacies, which pose a safety threat, with licensed website pharmacies, which probably sell good quality drugs. If this conclusion is replicated in other studies for other drugs and in larger sample sizes, then the safety issue will be clarified and will hopefully dissolve.

## Supporting Information

Appendix S1 How website pharmacies were selected.
(0.03 MB DOC)
