# Supplementary material for: Assessing Website Pharmacy Drug Quality: Safer Than You Think?
Source: PLoS One. 2010 Aug 13;5(8):e12199. doi: 10.1371/journal.pone.0012199 (PMC2921371; doi:10.1371/journal.pone.0012199)
Supplement: Appendix S1 — How website pharmacies were selected. (0.03 MB DOC) [file pone.0012199.s001.doc]

**APPENDIX**

**How website pharmacies were selected**

The authors selected website pharmacies in two ways (1) by using the NABP “recommended” and “not recommended” lists and (2) by using the two most heavily used search engines, Google and Yahoo!, to identify websites where each of the five brand name drugs could be purchased without a prescription, or where a prescription could be obtained via a questionnaire. For each drug, search terms included “[name of drug],” “cheap,” and “prescription” and “[brand name of drug].” The perspective was of a consumer looking for the cheapest drug available off-prescription.

Website pharmacies were classified into four broad groups:

1. **“Approved” (6 websites):** these pharmacies have been accredited by the NABP under their Verified Internet Pharmacy Practice Sites program, which is recognized by the U.S. FDA. From the list of 18 websites provided by NABP (list accessed in December 2008), drugs were purchased from six of them. The other twelve websites only sold drugs through insurance companies/membership organizations, required a prescription drug benefit card, or would not accept the author’s credit card after repeated attempts. All five drugs tested in this study were available from each website.
2. **“Legally Compliant” (10 websites)**: these pharmacies have not been accredited by NABP, but nor are they listed on NABP’s “not recommended” list. PharmacyChecker, an independent group not affiliated with any given pharmacy, indicates these websites are “approved” and in compliance with the laws in countries in which they are registered. **Google, Yahoo! and Microsoft** *require* all advertisers and their affiliates who sell prescription drugs to be approved by PharmacyChecker. From the list of twelve websites that the authors attempted to order from, drugs were only ordered from ten. Two websites that the authors attempted to order from would not accept prescriptions. All five drugs were available from seven of the ten websites.
3. **“Not Recommended”** **(10 websites)**: these pharmacies are listed as “approved” by PharmacyChecker, but are listed on NABP’s “not recommended” list. Drugs were procured from ten websites. All five drugs were only available from one of the websites. The authors attempted to purchase drugs from many other websites but encountered problems. Some only accepted Visa; others returned mailed-in or faxed prescriptions without explanation.
4. **“Highly Not Recommended” (15 websites):** these pharmacies are listed on NABP’s “not recommended” list and are unlisted or listed as “not approved” by PharmacyChecker. Drugs were procured from fifteen websites, none of which could provide all five drugs tested in this study. Some websites that were initially selected crashed when the lead author tried to order drugs; some only accepted Visa; others never delivered ordered (and paid for) drugs.
